# Nighttime Encounter

**DOI:** 10.5811/westjem.2015.6.27772

**Published:** 2015-07-13

**Authors:** Samuel Sampson

**Affiliations:** University of California Irvine, School of Medicine, Irvine, California

I felt the world narrow
as I looked at her, a frail woman
tonight thin and grey,
lent animus by memory and
desperation and loneliness.

She gestured weakly as I came to her
and said something, muffled,
under the thick, coiled tubing
which snaked to the mask
bound about her face.

I took the straps off,
and at this early morning hour
she straightened her hair feebly,
gazing in the darkness
as the machine sputtered and blew.

I rapidly ministered to it,
pleading for silence.

Freed now, she spoke;
Dimly, there arose an elegance,
rapidity and lucidity,
an English accent and gentle words
spilling out in her deprecating way.

She beckoned; I sat,
and held her hand.

She told me of her time -
she was a young woman,
on a boat, falling away,
journeying overseas, Australia,
to a new home far removed.

Of a man she had met there, and loved and buried.
Of her work -
she had thought it very important;
Of the children she had borne -
how she missed them.

For me, for herself,
she sketched the arc of her star.

Sitting on a precipice, she spoke:
What really mattered now, here
mostly alone, in the dark,
a small hospital room
and drawn curtains, fold-out fabric walls.

At times I held that blowing mask
against her face, to give her the breath,
at times I asked a question -
but mostly I listened
and held her hand.

The machine huffed, disconnected,
waiting, in the dark.

She smiled as she spoke,
sometimes mocking herself,
sometimes wry, sometimes happy,
on some things she couldn’t speak -
we both understood.

She held a strength, I knew,
found in those who dare reject hubris.

My pager interrupted,
it was the world interrupting, really,
I silenced it -
and sat with her and listened
until I could no more.

Her last words,
said with a smile:
“I know you’re busy.
Thank you for listening
to an old windbag like me.”

I told her it was my pleasure -
I have always honored teachers.

I strapped the mask back on
and smiled at her, constrained,
buried now, under mask and tubing
with life and machine
connected again, far from equal.

She gazed up at me, still and silent.
I gave her hand a squeeze,
and left into the world of light,
and movement,
and things to be done.

My world had expanded,
but it was her last conversation.

